# Water-Soluble *Saccharina latissima* Polysaccharides and Relation of Their Structural Characteristics with *In Vitro* Immunostimulatory and Hypocholesterolemic Activities

**DOI:** 10.3390/md21030183

**Published:** 2023-03-16

**Authors:** Ana S. P. Moreira, Diana Gaspar, Sónia S. Ferreira, Alexandra Correia, Manuel Vilanova, Marie-Mathilde Perrineau, Philip D. Kerrison, Claire M. M. Gachon, Maria Rosário Domingues, Manuel A. Coimbra, Filipe M. Coreta-Gomes, Cláudia Nunes

**Affiliations:** 1 LAQV-REQUIMTE—Associated Laboratory for Green Chemistry of the Network of Chemistry and Technology, Department of Chemistry, University of Aveiro, Campus Universitário de Santiago, 3810-193 Aveiro, Portugal; 2 i3S—Institute for Research and Innovation in Health and IBMC—Institute for Molecular and Cell Biology, University of Porto, 4200-135 Porto, Portugal; 3 ICBAS—Instituto de Ciências Biomédicas de Abel Salazar, Universidade do Porto, 4050-313 Porto, Portugal; 4 Scottish Association for Marine Sciences, Scottish Marine Institute, Oban PA37 1QA, UK; 5 Hortimare BV, Altonstraat 25A, 1704 CC Heerhugowaard, The Netherlands; 6 Unité Molécules de Communication et Adaptation des Micro-Organismes (UMR 7245), Muséum National d’Histoire Naturelle, Centre National de la Recherche Scientifique (CNRS), 75005 Paris, France; 7 CESAM—Centre for Environmental and Marine Studies, Department of Chemistry, University of Aveiro, Campus Universitário de Santiago, 3810-193 Aveiro, Portugal; 8 CQC-IMS—Coimbra Chemistry Centre, Institute of Molecular Sciences, University of Coimbra, 3004-535 Coimbra, Portugal; 9 CICECO—Aveiro Institute of Materials, Department of Materials and Ceramic Engineering, University of Aveiro, Campus Universitário de Santiago, 3810-193 Aveiro, Portugal

**Keywords:** seaweed, Laminariales, sulphated polysaccharides, fucans, fucoidans, mice, lymphocytes, flow cytometry, hypocholesterolemic effect, NMR

## Abstract

Brown macroalgae are an important source of polysaccharides, mainly fucose-containing sulphated polysaccharides (FCSPs), associated with several biological activities. However, the structural diversity and structure–function relationships for their bioactivities are still undisclosed. Thus, the aim of this work was to characterize the chemical structure of water-soluble *Saccharina latissima* polysaccharides and evaluate their immunostimulatory and hypocholesterolemic activities, helping to pinpoint a structure–activity relationship. Alginate, laminarans (F1, neutral glucose-rich polysaccharides), and two fractions (F2 and F3) of FCSPs (negatively charged) were studied. Whereas F2 is rich in uronic acids (45 mol%) and fucose (29 mol%), F3 is rich in fucose (59 mol%) and galactose (21 mol%). These two fractions of FCSPs showed immunostimulatory activity on B lymphocytes, which could be associated with the presence of sulphate groups. Only F2 exhibited a significant effect in reductions in *in vitro* cholesterol’s bioaccessibility attributed to the sequestration of bile salts. Therefore, *S. latissima* FCSPs were shown to have potential as immunostimulatory and hypocholesterolemic functional ingredients, where their content in uronic acids and sulphation seem to be relevant for the bioactive and healthy properties.

## 1. Introduction

Macroalgae (also known as seaweed) represent a sustainable source of natural bioactive compounds with potential for the development of new products and biomaterials to improve human health. These include compounds, such as polysaccharides, proteins, lipids, and pigments, which have great potential for commercial exploitation in the food, nutraceutical, pharmaceutical, and cosmetic industries [1,2].

Polysaccharides are major constituents of macroalgae, acting as structural components of cell walls and energy storage compounds. Brown macroalgae (Phaeophyceae) synthesize unique polysaccharides, which are the following: alginates, laminarans, and fucose-containing sulphated polysaccharides (FCSPs) [3]. Together, these polysaccharides could represent more than 50% of the dry weight of brown macroalgae [4]. Alginates are used for many commercial applications, mainly in food as thickening, gelling, and emulsifying agents [5]. Specifically, FCSPs have been subject of intensive research due to their biological activities and health-promoting functions [6]. However, alginates [7] and laminarans [8] have also been recognized for their bioactive properties, such as immunomodulatory, anti-obesity, and anti-diabetic.

Structurally, alginates are copolymers composed of (β1→4)-d-mannuronic acid (M), (α1→4)-l-guluronic acid (G), and alternating (MG) blocks. Laminarans, also known by their old name laminarins [9], are storage β-glucans and the most abundant glucose polysaccharides in brown seaweed, which also contain cellulose [10]. The laminarans are composed of a backbone of (β1→3)-linked d-glucose with branches of (β1→6)-linked d-glucose. Depending on their reducing ends, laminarans are classified as G chains (end with a glucose residue) and M chains (end with a mannitol) [4]. FCSPs in brown macroalgae represent a structurally diverse and complex fraction, including fucans and fucoidans. As a common structural feature, FCSPs are composed of sulphated α-l-fucose residues, which can be in the main backbone and/or side chains. The terms “fucans” and “fucoidans” are still misused in the literature. Fucans are polysaccharides with a backbone of fucose residues. Most of the fucans found in brown macroalgae have a backbone of (α1→3)- or alternating (α1→3)- and (α1→4)-linked residues of l-fucose, classified, respectively, as type I and type II. Fucoidans are heteropolysaccharides, presenting diverse backbones, which include other monosaccharides, such as uronic acids, galactose, mannose, and xylose. Acetyl groups have also been described in FCSPs [11,12].

In the case of brown seaweed *Saccharina latissima*, the main FCSPs are fucans composed of a backbone of (α1→3)-linked fucose residues sulphated at C4 and/or C2 and branched at C2 by a single sulphated fucose residue [13]. More recently, the presence of fucans in *S. latissima* was also reported, consisting of a backbone of (1→3)-linked fucose residues with (1→4)-linked fucose branches [14]. Moreover, other structures of FCSPs are described for *S. latissima*, including the following three types of fucoidans: (1) fucogalactans having a backbone of (β1→6)-linked d-galactose residues branched mainly at C4 and containing both terminal galactose and fucose residues; (2) fucoglucuronans with a backbone of (β1→3)-linked glucuronic acid residues branched at C4 by single fucose residues; and (3) fucoglucuronomannans composed of a backbone of alternating (β1→4)-linked d-glucuronic acid and (α1→2)-linked d-mannose and branches of single fucose residues at C3 of mannose [13].

FCSPs (mostly referred as fucoidans) have been associated with several biological activities, including anticoagulant, anti-inflammatory, antiviral, antitumor, immunomodulatory [4,15,16], and hypocholesterolemic activity [17]. The biological properties of FCSPs depend on their structural details, such as molecular size, degree of sulphation, and constituent monosaccharides [15,18]. Further, it is known that the content and composition of FCSPs are variable between brown seaweed species [19,20], as well as the distinct environmental conditions associated with different geographical locations and harvesting seasons [10,21,22]. The composition of identified FCSPs is also dependent on the methods used for extraction and purification [19]. However, there is no standard protocol for the separation of these fractions [23], and the structural diversity and heterogeneity of the FCSPs impair the isolation of fucans/fucoidans in pure and homogeneous fractions [24].

Among the most commercially relevant brown macroalgae [25], *S. latissima* (previously named as *Laminaria saccharina*) is native to the North Atlantic and North Pacific [10]. *S. latissima*, commonly known as sugar kelp, is listed in European Union novel food catalogue, a list of authorized novel foods safe for human consumption [25]. As natural stocks are limited, the collection of *S. latissima* from the wild needs to be restricted to maintain the integrity of coastal marine ecosystems. Indeed, cultivation of *S. latissima* has been developed to increase stocks for commercial uses, namely with European funding (e.g., GENIALG project). However, a deeper knowledge on the *S. latissima* bioactive compounds is still needed to boost their added value and potential applications. Specifically, an understanding of the structures of its polysaccharides (mainly of FCSPs) and their bioactivities is far from being known.

In this context, this work aims to characterize the chemical structure of water-soluble *Saccharina latissima* polysaccharides and evaluate their immunostimulatory and hypocholesterolemic activities. *S. latissima* polysaccharides were selected for this work considering previous studies that reported bioactive properties of FCSPs and laminarans extracted from other brown macroalgae, namely of *Saccharina* genus (*S*. *japonica* and *S. sculpera*) [26,27,28,29]. In addition, unfractionated hot water extract of *S. latissima* showed immunomodulatory properties towards human THP-1-derived macrophages [30]. Herein, for the first time, the potential of fractionated water-soluble *S. latissima* polysaccharides for immunostimulatory and hypocholesterolemic purposes was assessed, helping to pinpoint a structure–activity relationship. For that, polysaccharides from cultivated *S. latissima* were extracted and fractionated using a green solvent-based procedure, including ethanol extraction of non-polysaccharide compounds, hot water extraction, alginate precipitation with calcium chloride, and anion exchange chromatography. In addition to the chemical characterization, polysaccharide-enriched fractions of *S. latissima* were tested for their *in vitro* lymphocyte stimulatory activity using BALB/c mice splenocytes. Their effects on cholesterol solubility were also evaluated using a simplified *in vitro* model composed of glycodeoxycholic acid (GDCA) bile salt.

## 2. Results and Discussion

### 2.1. Fractionation and Characterization of S. latissima Polysaccharides

**Figure 1:**
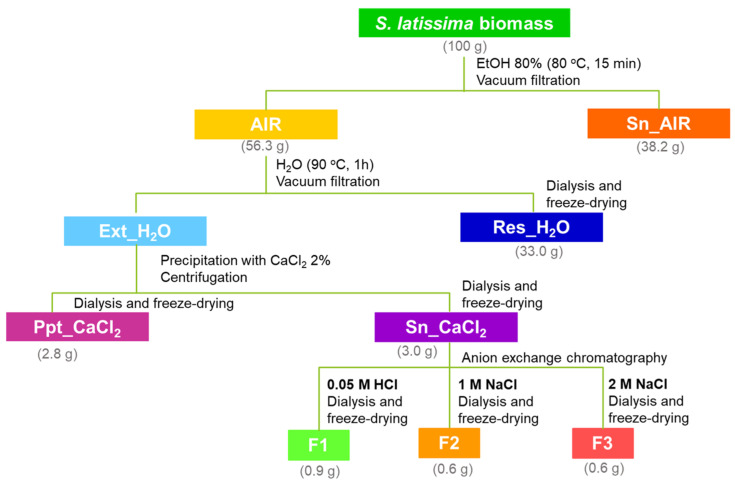
Procedure used for extraction and fractionation of *S. latissima* polysaccharides. Values in parenthesis indicate the yield from 100 g of freeze-dried biomass.

The hot-water-soluble polysaccharides of *S. latissima* were extracted from the alcohol-insoluble residue (AIR) obtained with 80% ethanol. Afterwards, alginate was separated via precipitation with CaCl_2_ (Ppt_CaCl_2_) and the polysaccharides present in the supernatant (Sn_CaCl_2_) were further fractionated by anion-exchange chromatography (Figure 1). The *S. latissima* biomass and all the fractions were characterized by total sugars content and monosaccharide composition (Table 1).

*S. latissima* biomass accounted for 42.0% (*w*/*w*) of total sugars. Uronic acids (UA, 37.3 mol%), mannose (Man, 29.9 mol%), and glucose (Glc, 25.6 mol%) were the main sugars, followed by fucose (Fuc, 4.2 mol%), galactose (Gal, 2.1 mol%), and xylose (Xyl, 0.9 mol%). Glc is mainly associated with the presence of glucose-rich polysaccharides (laminarans and cellulose), whereas Fuc and Gal are components of fucose-containing sulphated polysaccharides (FCSPs). UA should mostly derive from alginates but may also be constituents of FCSPs. Most of the Man detected may derive from free mannitol, which occurs naturally in Phaeophyta (including *S. latissima*) and has a recognized role in osmotic regulation [31]. Therefore, the sugars composition corroborated the presence of different polysaccharides in *S. latissima*, as reported for this species [10] or brown seaweed in general [11].

Mannitol and mannose were previously identified (by HPAEC-PAD) in dried biomass of *S. latissima* from Iceland in a proportion of 2:1, together accounting for about 3% [19]. In the same biomass (total sugars of 68%), UA represented about 47% of *S. latissima* biomass, including mannuronic acid (36%) and guluronic acid (9%), components of alginate, and glucuronic acid (2%) [19]. The differences in monosaccharide composition and content of our results compared with the results from the literature may be related to the geographical origin and harvesting time, as well as to different conditions of macroalgae processing (i.e., washing step) and experimental procedures (i.e., hydrolysis step). Specifically, *S. latissima* is known to have wide seasonal variation in the content of mannitol (0.5–24%) [10].

In this work, the extraction with ethanol was carried out to eliminate non-polysaccharide components, such as lipids [32], pigments [33], and mannitol [31], from macroalga biomass. The AIR, containing the *S. latissima* polysaccharides, was recovered with a yield of 56.3% (*w/w* of macroalga biomass), whereas supernatant (Sn_AIR), containing the non-polysaccharide components, represented 38.2%. The AIR had a total sugars content of 53.4% and contained UA (59.8 mol%, Glc (26.0 mol%), and Fuc (6.8 mol%) as main sugars. The Sn_AIR comprised 24.9% of total sugars, of which Man (91.2 mol%) was predominant. The Man identified in Sn_AIR may derive from naturally occurring mannitol [31], which was extracted from macroalga biomass with 80% ethanol.

Hot-water extraction from AIR allowed us to obtain a residue (Res_H_2_O) with a yield of 58.7% (w/w of AIR). This residue was composed of 50.8% of sugars, of which UA (64.1 mol%) and Glc (25.2 mol%) were the most abundant. The hot-water extract (Ext_H_2_O; 53.5% of total sugars) revealed a composition of similar amounts of Glc (35.3 mol%) and UA (33.4 mol%), followed by Man (15.1 mol%) and Fuc (11.4 mol%). The higher percentage of Fuc found in Ext_H_2_O than in Res_H_2_O suggests that most FCSPs are soluble in water at 90 °C. This is also corroborated by the amount of Man in Ext_H_2_O, as Man is also described as a constituent of FCSPs. The Glc found in the hot-water-insoluble fraction (Res_H_2_O) was probably derived from cellulose present in *S. latissima* cell walls, whereas the Glc found in the soluble one (Ext_H_2_O) most likely derived from laminarans [10].

#### 2.1.1. Precipitation with Calcium Chloride

**Figure 2:**
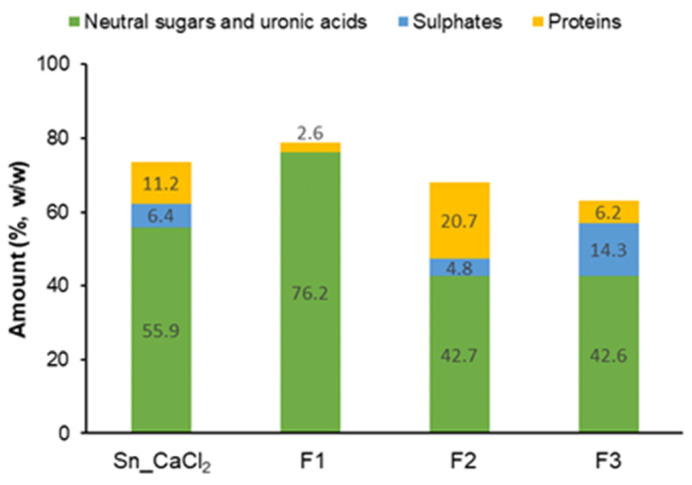
Content (%, *w*/*w*) of sugars (neutral and uronic acids), sulphates (calculated as -SO_3_^−^), and proteins found in polysaccharide-enriched fractions obtained from *S. latissima* (Sn_CaCl_2_, F1, F2, and F3). Mean values are represented. Mean ± SD are presented in Table 1 (for sugars) and Supplementary Table S1 (for sulphates and proteins).

As UA found in Ext_H_2_O may derive in part from alginate, calcium chloride (CaCl_2_) was added to the Ext_H_2_O to separate this polysaccharide by precipitation. The precipitate recovered after centrifugation (Ppt_CaCl_2_) had 62.1% of sugars, mostly UA (93.5 mol%), corroborating the presence of alginates. Further, minor amounts of neutral sugars, mainly Fuc (3.8 mol%), were identified in Ppt_CaCl_2_, possibly due to a small proportion of co-precipitated FCSPs. Glc (47.7 mol%) and Fuc (23.7 mol%) were the main sugars found in the supernatant (Sn_CaCl_2_), probably due to the presence of laminarans and FCSPs, respectively. Total sugars (neutral and UA) accounted for 55.9% of the Sn_CaCl_2_ fraction, whereas sulphate esters (as -SO_3_^−^) represented 6.4% and proteins represented 11.2% (Figure 2 and Supplementary Table S1).

#### 2.1.2. Anion-Exchange Chromatography

**Figure 3:**
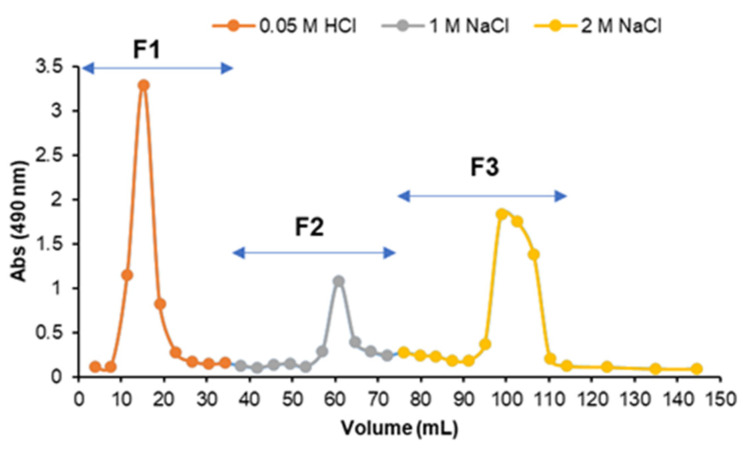
Anion-exchange chromatography on DEAE-Trisacryl M of Sn_CaCl_2_ fraction, with stepwise elution using 0.05 M HCl (F1), 1 M NaCl (F2), and 2 M NaCl (F3). Elution profile was monitored by phenol-sulfuric acid method (490 nm). No signal was observed with 4 M NaCl used for column cleaning.

To fractionate the polysaccharides present in Sn_CaCl_2_, this fraction was subjected to anion-exchange chromatography, recovering three fractions with increasing ionic strength: F1 (eluted with 0.05 M HCl), F2 (eluted with 1 M NaCl), and F3 (eluted with 2 M NaCl) (Figure 3).

Fraction F1, eluted without ionic strength, had a total content of sugars of 76.2%, mainly composed of Glc (97.7 mol%) (Table 1). No sulphate was detected in this fraction and protein was found in low amounts (2.6%) (Figure 2). Considering the neutral character and sugars composition, it can be confirmed that F1 is a laminaran-enriched fraction. The low amount of Man (2.3 mol%) found in F1 may derive from mannitol located at the reducing end of laminarans (classified as M chains) [10].

Fraction F2, eluted with the lowest ionic strength, was composed of 42.7% of sugars. UA (44.7 mol%) and Fuc (28.7 mol%) were the main sugars, together with minor amounts of Gal (8.5 mol%), Glc (6.8 mol%), Xyl (5.7 mol%), and Man (5.6 mol%) (Table 1). Sulphate content (as SO_3_^−^) represented 4.8% of this fraction. In addition, F2 also had 20.7% of protein (Figure 2).

The fraction F3, eluted with the strongest ionic strength, showed a total content of sugars of 42.6%. F3 was mainly composed of Fuc (59.1 mol%), Gal (20.8 mol%), and UA (12.0 mol%), containing also Glc (3.2 mol%), Xyl (3.0 mol%), and Man (2.0 mol%) (Table 1). Sulphates accounted for 14.3% of F3 and a low content of protein was detected in this fraction (6.2%) (Figure 2).

F2 and F3 are not pure/homogeneous fractions of FCSPs, which is in accordance with previous studies on FCSPs of *S. latissima* that also used anion-exchange chromatography for fractionation [13,19]. F2 is possibly a complex mixture, containing mainly (β1→3)-glucuronan chains [13]. F3 fraction probably contains mainly sulphated fucans, among other polysaccharide structures [13]. As described in the literature, FCSPs are complex heteropolysaccharides composed of several types of monosaccharides that, due to the presence of uronic acid residues and sulphate groups, are highly charged. Regarding the protein content found in F2 and F3, other studies also reported the presence of proteins in FCSP-enriched fractions of brown macroalgae (including *S. latissima*), suggesting that proteins are tightly associated with certain FCSPs [34,35].

### 2.2. In Vitro Lymphocyte Stimulatory Activity

The selected fractions Sn_CaCl_2_, Ppt_CaCl_2_, F1, F2, and F3, containing different proportions of water-soluble polysaccharides from *S. latissima* (alginate, laminarans, and FCSPs), were incubated with murine splenocytes to evaluate *in vitro* lymphocyte stimulatory activity. For all the fractions and concentrations tested (25, 100, and 250 µg/mL), the cell viability was not significantly decreased when compared to the negative control with cells cultured only with medium. The same occurred in the presence of polymyxin B (PB), used to assess possible endotoxin contamination (Supplementary Figure S1).

The percentage of T cells activated in the presence of polysaccharide-enriched fractions from *S. latissima* ranged from 1.3 to 4.2% for all tested concentrations, inferred by the expression of the early activation marker CD69 on the surface of CD3^+^ cells (Supplementary Figure S2). Only the Sn_CaCl_2_ fraction (mixture of FCSPs and laminarans) at 250 µg/mL induced significant T-cell activation when compared to the negative control (4.2% vs. 1.4%) but negligible when compared to concanavalin A (ConA) used as a positive control of T-cell activation (84.4%) (Supplementary Figure S2).

**Figure 4:**
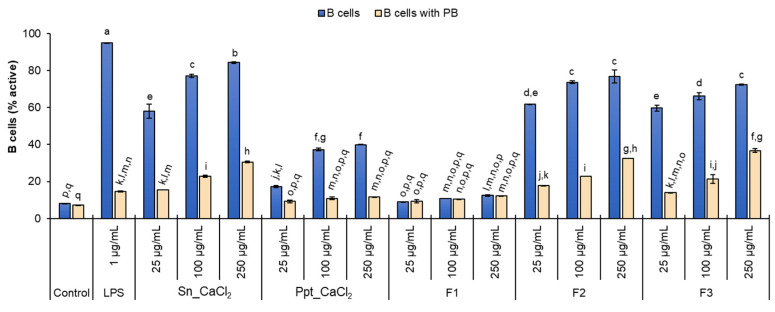
Percentage of B cells activated by incubation with polysaccharide-enriched fractions obtained from *S. latissima* (Sn_CaCl_2_, Ppt_CaCl_2_, F1, F2, and F3) at concentrations of 25, 100, and 250 µg/mL, in the absence and presence of polymyxin B (PB). Culture medium alone (RPMI) and incubation with lipopolysaccharide (LPS) was used as negative and positive control, respectively. Mean (±SD) values are represented. Different letters above the bars indicate statistically significant differences between compared groups (*p* < 0.05).

On the other hand, CD19^+^ cells (B cells) were significantly stimulated by all fractions, except for F1 (laminaran-enriched fraction). The B-cell activation occurred in a dose-dependent manner. The percentage of B cells expressing CD69 (8.0% in negative control) increased upon incubation with the different polysaccharide concentrations used, ranging from 25 to 250 µg/mL, as follows: 58.1% to 84.4% for Sn_CaCl_2_ (containing FCSPs and laminarans); 17.3% to 39.9% for Ppt_CaCl_2_ (mainly alginates); 61.8% to 76.9% for F2 (moderate charged FCSPs); and 59.7% to 72.4% for F3 (high charged FCSPs) (Figure 4).

In addition, the percentage of B cells activated by incubation with *S. latissima* fractions was evaluated in the presence of polymyxin B (PB) to assess possible contamination of the samples with bacterial endotoxin (LPS). PB is known to bind LPS and inhibit LPS-induced B-cell activation [36,37]. Indeed, the treatment of cells with PB reduced B-cell activation induced by LPS (used as positive control) from 95.0% to 14.6%. PB also reduced the extent of B-cell activation in cultures stimulated with *S. latissima* fractions, indicating that LPS contamination cannot be completely excluded. However, even in the presence of PB and for the three concentrations tested, the percentage of B cells activated by incubation with fractions containing FCSPs (Sn_CaCl_2_, F2, and F3) remained significantly higher than the negative control (7.3%). This effect was also dose-dependent, increasing from 25 to 250 µg/mL of polysaccharides, as follows: 15.5% to 30.6% (Sn_CaCl_2_), 17.7% to 32.6% (F2), and 14.0% to 36.7% (F3) (Figure 4). Moreover, the activation percentages found for cells stimulated with 100 and 250 µg/mL of Sn_CaCl_2_, F2, and F3 in the presence of PB were significantly higher than those observed for LPS stimulation with PB.

Considering that F1 (only containing laminarans) did not exhibit immunostimulatory activity on B lymphocytes, the activity of Sn_CaCl_2_, F2, and F3 can be directly associated with the presence of FCSPs. These results are in line with a previous study comparing fucoidans and laminarans extracted from other Laminariaceae species (*Laminaria japonica*), where fucoidans exhibited a stronger immune activation ability [29]. The co-precipitation of FCSPs in Ppt_CaCl_2_ (enriched in alginates) may contribute to the immunostimulatory activity on B lymphocytes observed in this fraction. However, studies have shown that alginates (including oligomer derivatives) themselves have immunostimulating effects, namely by inducing B cells to express CD69 [38]. Even so, the B-cell activation induced with Ppt_CaCl_2_ was significantly lower than that observed for Sn_CaCl_2_, F2, or F3 fractions, suggesting that FCSPs have a higher activation capacity than alginates. The presence of sulphate esters in the polysaccharides, as occurs in FCSPs, has been reported to be relevant for immunostimulatory activity [39,40]. On the other hand, co-extracted laminarans in Sn_CaCl_2_ had no negative impact on B-cell activation promoted by FCSPs. The possibility of eliminating the fractionation step of the Sn_CaCl_2_ may be advantageous considering potential applications, namely in the formulation of functional foods or nutraceuticals.

Regarding the importance of lymphocyte activation, it is worth noting that B cells are found along the intestinal tract in Peyer’s patches. This is a possible route for direct B-cell activation by FCSPs, as hypothesized for other polysaccharides [41]. Indeed, intestinal immunomodulating activity via Peyer’s patch cells was reported for fucoidans extracted from two brown seaweed species (*Sargassum crassifolium* and *Padina australis*) [42]. Another hypothesis is indirect B-cell activation promoted by cytokines produced by polysaccharide-stimulated enterocytes or phagocytes [41].

### 2.3. In Vitro Hypocholesterolemic Effect

The effect of polysaccharide-enriched fractions obtained from *S. latissima* (Sn_CaCl_2_, F1, F2, and F3) in the sequestration of GDCA bile salt and in reductions in cholesterol solubilized in GDCA micelles was evaluated by quantitative NMR and compared with cationic resin colestipol used as a positive control (Supplementary Figure S3).

**Figure 5:**
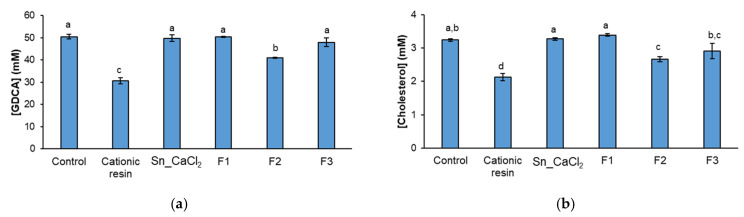
Concentration of (**a**) bile salts (GDCA) that remained in solution and (**b**) cholesterol solubilized in the presence of polysaccharide-enriched fractions obtained from *S. latissima* (Sn_CaCl_2_, F1, F2, and F3). Model with GDGA and cholesterol was used as negative control and cationic resin was used as positive control. Mean (±SD) values are represented. Different letters above the bars indicate statistically significant differences between compared groups (*p* < 0.05).

The F2 fraction, containing FCSPs eluted with a lower ionic strength in the anion-exchange chromatography, significantly decreased the amount of GDCA in solution when compared to the negative control (GDCA with cholesterol), whereas no significant differences were observed for Sn_CaCl_2_ (containing neutral laminarans and charged FCSPs), F1 (laminarans), and F3 (containing highly charged FCSPs) (Figure 5a). In addition, cholesterol solubility decreased significantly in the presence of fraction F2 when compared to the negative control (Figure 5b), being coincident with a decrease in bile salt concentration. Indeed, the quantity of solubilized cholesterol in the presence of F2 was proportional to the amount of GDCA in solution (Figure 5a,b). A chemical shift to lower ppm was also noticed in bile salt resonances, being evidence of the interaction between bile salt and the polysaccharide (Supplementary Figure S3). This corroborates that the mechanism behind reductions in cholesterol solubilization is the sequestration of bile salts by moderately charged FCSPs. Considering its composition, the F2 fraction is rich in uronic acids but has a lower content of fucose and sulphate groups in comparison with F3. Therefore, uronic acids (contrarily to sulphate groups) may have an important role on GDCA sequestration. Other types of polysaccharides primarily composed of uronic acids have been reported to interact with bile acids [43,44], namely pectins, with a degree of methylesterification of 62% [43]. Considering that GDCA is negatively charged at the intestinal lumen pH, the interactions behind the negatively charged polysaccharides and bile salts should be mostly due to hydrophobic interactions, possible involving the fucose (a deoxy sugar) present in FCSPs. 

In the present work, laminarans (F1) from *S. latissima* showed no effect, either in the sequestration of GDCA or cholesterol solubility. Although other β-glucans from cereals (barley and oat) and mushroom have been known to sequestrate bile salts and decrease cholesterol solubility, their structure is different from brown macroalgae laminarans. Cereal β-glucans have (β1→3)- and (β1→4)-Glc linkages. Both laminarans and mushroom β-glucans are composed of (β1→3) and (β1→6)-Glc, but laminarans have a reported lower average molecular weight [44]. This structural difference could explain the absence of a hypocholesterolemic effect of laminarans. 

Like colestipol, a cationic resin used as a cholesterol-lowering drug, FCSPs from the F2 fraction seem to have the capacity (albeit to a lesser effect) to sequester bile salts, reducing their concentration in the intestinal lumen, lowering the cholesterol solubility, and limiting cholesterol absorption through the intestine. Considering other brown macroalgae, the fraction of *Sargassum zhangii* containing moderately charged FCSPs (eluted with 1 M NaCl) showed the best ability to bind bile acids and reduce the content of intracellular total cholesterol in HepG2 cells compared to those eluted with 0.5 M or 2 M NaCl [45]. These data also corroborate the higher potential of moderately charged FSCPs as hypocholesterolemic agents able to decrease cholesterol absorption, which is especially relevant considering the prevalence of the high cholesterol levels in human blood, a well-established risk factor for cardiovascular diseases [46].

## 3. Materials and Methods

### 3.1. Extraction and Fractionation of S. latissima Polysaccharides

The macroalga *S. latissima* was cultivated by the Scottish Association for Marine Science (Oban, UK) at the Port a’ Bhuiltin seaweed farm (56.4886° N, −5.4698° E) and harvested in May 2017 [32]. Freeze-dried and milled biomass was used for extraction and fractionation of *S. latissima* polysaccharides, as represented in Figure 1.

#### 3.1.1. Alcohol-Insoluble Residue (AIR) Preparation

*S. latissima* biomass (20 g) was suspended in distilled water (160 mL) and kept under stirring for 10 min at room temperature. Then, absolute ethanol (640 mL) was added, and the mixture was left for 15 min at 80 °C. The solution was filtered with a funnel with porous plate and a 110 nm filter under vacuum. The alcohol-insoluble residue (AIR) was washed with ethanol and acetone and left in the hood overnight to dry. The filtrate (Sn_AIR) was also recovered, concentrated on rotary evaporator at 40 °C to remove ethanol, and freeze-dried.

#### 3.1.2. Hot Water Extraction

To extract hot-water-soluble polysaccharides, AIR (5 g) was suspended in distilled water (560 mL) and then kept at 90 °C for 1 h under stirring. An extract (Ext_H_2_O) and a residue (Res_H_2_O) were recovered by filtration under vacuum. Res_H_2_O and an aliquot of the Ext_H_2_O (1 mL) were dialyzed (cut-off 12 kDa) against distilled water and freeze-dried for further analysis.

#### 3.1.3. Precipitation with Calcium Chloride

For alginate precipitation, CaCl_2_ 2% (3.8 g) was added to the Ext_H_2_O recovered by filtration (with a volume of 380 mL) and stirred for 25 min at room temperature. The solution was kept for 2 h at 4 °C and then centrifuged at 15,000 rpm for 20 min at 4 °C. The resulting precipitate (Ppt_CaCl_2_) and supernatant (Sn_CaCl_2_) were dialyzed and freeze-dried.

#### 3.1.4. Anion-Exchange Chromatography of Fraction Sn_CaCl_2_

Sn_CaCl_2_ fraction (100 mg) was dissolved in 2 mL of 0.05 M HCl and applied to a DEAE-Trisacryl M (Sigma-Aldrich, St. Louis, MO, USA) column (15 cm × 1.6 cm internal diameter), pre-equilibrated with 0.05 M HCl and an adjusted flux of 0.5 mL/min. The retained material on the column was eluted with a stepwise elution with solutions increasing ionic strength. A first fraction was eluted with 0.05 M HCl (F1) and collected until reaching a total volume of 35 mL. Two fractions (40 mL each) were then recovered by elution with 1 M NaCl (F2) and 2 M NaCl (F3). Each fraction (F1, F2 and F3) was dialyzed and freeze-dried. A solution of 4 M NaCl was used for column cleaning. The elution profile was obtained by recovery of the fractions (1.9 mL), which were assessed by the colorimetric phenol-sulfuric acid method [47].

### 3.2. Neutral Sugars Analysis and Uronic Acid Determination

Polysaccharide fractions (2 mg) were subjected to pre-hydrolysis for 3 h at room temperature with 72% H_2_SO_4_ (*w*/*w*), followed by hydrolysis at 100 °C with 1 M H_2_SO_4_ for 2.5 h. Neutral sugars were determined by converting the hydrolyzed sugars in alditol acetates, as described previously [48], using 2-desoxyglucose as an internal standard. Alditol acetates were analyzed on a GC-FID (Perkin-Elmer Clarus 400, Waltham, MA, USA) equipped with DB-225 column (30 m of length, 0.25 mm of internal diameter and 0.15 µm of film thickness; Agilent J&W GC columns, Santa Clara, CA, USA), operating with injector temperature of 220 °C, detector temperature of 230 °C, and hydrogen flow rate of 1.7 mL/min. The oven was programmed as follows: 200 °C (held for 1 min), 40 °C/min to 220 °C (held for 7 min), and 20 °C/min to 230 °C (held for 1 min).

For uronic acid (UA) determination, the polysaccharide fractions were hydrolyzed for 1 h with 1 M H_2_SO_4_ at 100 °C. Uronic acids were estimated by colorimetry using phenol-sulfuric acid method using d-galacturonic acid as standard, as previously detailed [48].

### 3.3. Sulphate and Protein Content

Sulphur (S) and nitrogen (N) contents of the fractions Sn_CaCl_2_, F1, F2, and F3 (2 mg per replicate) were determined by elemental analysis (Leco Truspec-Micro CHNS 630-200-200 elemental analyser) [48]. Considering the determined sulphur content, the content of sulphate groups (calculated as -SO_3_^−^) was estimated. The protein content was obtained using nitrogen–protein conversion factor of 4.37, a specific factor determined for *S. latissima* [49].

### 3.4. In Vitro Lymphocyte Stimulatory Activity

BALB/c mice were purchased from Charles River (Barcelona, Spain) and kept at i3S animal facilities. Spleen cell suspensions were obtained from naïve mice included in the project licensed by the competent national authority (Direção Geral de Alimentação e Veterinária, Lisbon, Portugal) with the reference number 001879/2021-01-06. The preparation of spleen lymphocytes for stimulation assays and analysis by flow cytometry were conducted as previously described [37,50]. Spleens were aseptically removed and splenocyte suspensions were obtained by mechanically disrupting the organ in Hanks’ balanced salt solution (HBSS, Sigma, St. Louis, MO, USA) and filtering through 100 μm cell strainers. Splenocytes were resuspended in ammonium-chloride-potassium (ACK) lysing buffer for 3 min, to lyse erythrocytes, washed with HBSS, and resuspended in RPMI-1640 medium (Sigma, St. Louis, MO, USA) supplemented with 10% foetal calf serum (Biowest, Nuaillé, France), 10 mM HEPES solution (Sigma), 100 IU/mL penicillin (Sigma, St. Louis, MO, USA), 50 mg/L streptomycin (Sigma, St. Louis, MO, USA), and 50 nM 2-mercaptoethanol (Merk, Darmstadt, Germany) (RPMI). Spleen cell suspensions were distributed in 96-well plates (10^6^ cells/well) and cultured for 6 h at 37 °C in a humidified atmosphere containing 5% CO_2_. Cells were stimulated with RPMI medium alone (negative control), 2.5 µg/mL of bacterial lipopolysaccharide (LPS) from *Escherichia coli* O111:B4 (Sigma, St. Louis, MO, USA; B cell positive control), 2.5 µg/mL of concanavalin A (Sigma, St. Louis, MO, USA; T cell positive control), or with one of the selected *S. latissima* fractions (Sn_CaCl_2_, Ppt_CaCl_2_, F1, F2, and F3) at 25, 100, and 250 µg/mL (of total sugars). Co-incubation with 100 µg/mL polymyxin B (PB; Sigma, St. Louis, MO, USA) was also tested to assess possible contamination of the samples with bacterial LPS. Monoclonal antibodies used (diluted 1:100) were as follows: anti-CD19 (PE-conjugate; clone 1D3; Biolegend, San Diego, CA, USA), anti-CD3 (PE-Cy7-conjugate; clone 145-2C11; BD Biosciences, Franklin Lakes, NJ, USA), and anti-CD69 (FTIC-conjugate; clone H1.2F3; Biolegend, San Diego, CA, USA). Prior to analysis, 5 µg/mL propidium iodide (Sigma) was added to the samples. Lymphocytes were analyzed in a BD FACSCanto™ II flow cytometer using BD FACSDiva™ Software 6.3.1 version (BD Biosciences, Franklin Lakes, NJ, USA). Data analysis was performed using FlowJo™ software 10.8.1 version (Ashland, OR, USA). Gating strategy used in FACs analysis is shown in Supplementary Figure S4. 

### 3.5. In Vitro Assessment of Hypocholesterolemic Effect

To assess the potential of *S. latissima* polysaccharides in reductions in cholesterol’s solubility, mixtures of selected fractions (Sn_CaCl_2_, F1, F2, and F3) were tested using an *in vitro* intestinal model composed of a 50 mM bile salt sodium glycodeoxycholate (GDCA) and 3.5 mM [4-^13^C]Cholesterol, as described previously [51,52]. The solutions were prepared in aqueous buffer, containing 10 mM Tris-HCl (pH 7.4), 0.15 M NaCl, 1 mM EDTA, and 0.02% sodium azide (NaN_3_) in deuterated water (D_2_O) containing 3-(Trimethylsilyl)propionic-2,2,3,3-d4 acid sodium salt (TSP). Cationic resin colestipol was used as positive control at a similar concentration as the *S. latissima* extracts (5 mg/mL). Before measurements, the mixtures were left under stirring at 100 rpm and 37 °C for 24 h. ^13^C NMR spectra were acquired at 37 °C using a 90° pulse, with a 25,252 Hz spectral width, acquisition time of 1.3 s, relaxation delay of 5 s, and 2040 acquisition scans. Proton decoupling was accomplished by using a WALTZ-16 decoupling sequence. Nuclear Overhauser Enhancement (NOE) was obtained through the comparation between ^13^C spectra with full proton decoupling and with proton decoupling only during acquisition [52]. ^1^H NMR spectra were acquired with a 90° pulse, a 7500 Hz spectral width, acquisition time of 1 s, relaxation delay of 5 s, and 128 acquisition scans. These experiments were acquired in 500 MHz NMR Bruker spectrometer and spectra were treated using MestreNova 6.1.1 (Mestrelab Research, Santiago de Compostela, Spain). With this methodology, due to their size, polysaccharides with GDCA or cholesterol aggregates and cholesterol precipitated as crystals are not observed by liquid state NMR, because the motion of these aggregates leads to a line-width broadening resulting from spin–spin relaxation (T2) phenomena. Therefore, bile salt sequestration and cholesterol emulsified in GDCA micelles (small aggregates) were determined by quantitative ^13^C NMR using the area of TSP (10 mM) resonating at 0 ppm, as internal standard for quantification. The area of carbon 4 from ^13^C enriched cholesterol, which resonates at 44.4 ppm, was used for its quantification, normalized by ^13^C enrichment factor (1.109/99.8). Regarding bile salt GDCA, several resonances CH_3_, CH_2_, CH, and C areas were used for quantification by ^13^C NMR (assignments are shown in Supplementary Table S2). In Supplementary Figure S3, the resonance CH position 5 of GDCA (45.0 ppm) is highlighted in the insert. All the areas obtained in ^13^C NMR spectrum were corrected for the NOE effect, which was used to decrease the length of the ^13^C NMR experiment per assay, without compromising the quantitative outcome of the experiment. Crosscheck of the results obtained by ^13^C NMR with NOE correction factors was conducted by comparison with the results obtained by quantitative ^1^H NMR experiments in the case of GDCA. The same approach was not addressed for cholesterol because no distinctive resonance from cholesterol was assigned by ^1^H NMR.

### 3.6. Statistical Analysis

Statistical analysis was performed using Minitab 17. Data from *in vitro* assays were analyzed by one-way ANOVA, followed by Tukey’s test. The confidence level was set at 95% (α = 0.05).

## 4. Conclusions

The consumption of macroalgae has long been related with health benefits, which can be related to the presence of structurally different polysaccharides. In this work, two fractions of fucose-containing sulphated polysaccharides (FCSPs) of *Saccharina latissima* with distinct charge and composition revealed similar immunomodulatory activity on B lymphocytes. The presence of sulphate esters in the structure of FCSPs seems to be a key feature for its immunomodulatory activity. However, for the bile salt sequestration, the content in uronic acids in FCSPs seems to be more relevant. Only moderately charged FCSPs, which have a higher content in uronic acids but lower fucose and sulphate content, showed hypocholesterolemic potential, exhibiting the capacity to sequester bile salts and reduce cholesterol solubility. On other hand, laminarans of *S. latissima* showed no immunostimulatory or hypocholesterolemic effect. Overall, fucose-containing sulphated polysaccharides of brown macroalga *Saccharina latissima* showed immunostimulatory and hypocholesterolemic activities, having potential to be used as functional ingredients.

## Figures and Tables

**Table 1:** Yield, sugars composition (mol%), and total sugars content of macroalga *S. latissima* biomass and fractions obtained during fractionation.

Sample	Yield (%, *w*/*w*)	Sugars Composition (mol%)						Total Sugars (%, *w*/*w*)
		Fuc	Xyl	Man	Gal	Glc	UA	
Biomass	-	4.2 ± 1.1	0.9 ± 0.4	29.9 ± 6.5	2.1 ± 0.3	25.6 ± 9.4	37.3 ± 5.9	42.0 ± 9.5
Sn_AIR	38.2 ^a^	0.2 ± 0.1	ND	91.2 ± 1.1	1.7 ± 0.2	6.9 ± 0.9	ND	24.9 ± 0.2
AIR	56.3 ^a^	6.8 ± 1.2	1.6 ± 0.8	3.9 ± 1.4	1.9 ± 0.3	26.0 ± 2.6	59.8 ± 4.8	53.4 ± 6.6
Hot-water extraction of AIR								
Res_H_2_O	58.7 ^b^	3.6 ± 0.1	1.1 ± 0.1	4.6 ± 0.8	1.5 ± 0.3	25.2 ± 0.9	64.1 ± 2.2	50.8 ± 2.8
Ext_H_2_O ^d^	-	11.4	1.6	15.1	3.2	35.3	33.4	53.5
Precipitation with calcium chloride of Ext_H_2_O								
Ppt_CaCl_2_	5.0 ^b^	3.8 ± 0.3	ND	1.9 ± 0.2	ND	0.7 ± 0.03	93.5 ± 0.5	62.1 ± 3.3
Sn_CaCl_2_	5.4 ^b^	23.7 ± 2.1	2.5 ± 0.4	1.5 ± 0.1	6.7 ± 0.7	47.7 ± 3.4	17.9 ± 1.6	55.9 ± 5.0
Anion-exchange chromatography of Sn_CaCl_2_								
F1	28.6 ^c^	ND	ND	2.3 ± 1.9	ND	97.7 ± 1.9	tr	76.2 ± 3.4
F2	19.3 ^c^	28.7 ± 0.1	5.7 ± 0.2	5.6 ± 0.2	8.5 ± 0.2	6.8 ± 0.6	44.7 ± 0.3	42.7 ± 4.8
F3	18.4 ^c^	59.1 ± 2.7	3.0 ± 1.1	2.0 ± 0.6	20.8 ± 4.2	3.2 ± 1.6	12.0 ± 2.1	42.6 ± 7.2

^a^ Results are expressed as weight % of macroalga biomass; ^b^ Results are expressed as weight % of AIR; ^c^ Results are expressed as weight % of Sn_CaCl_2_; ^d^ Results for a single aliquot taken before precipitation with calcium chloride. ND, not detected. tr, traces.

## Data Availability

Data is contained within the article.

## Supplementary Materials

The following supporting information can be downloaded at: https://www.mdpi.com/article/10.3390/md21030183/s1, Table S1: Content (%, *w*/*w*) of sulphur (S) and nitrogen (N) determined by elemental analysis, as well as sulphates (calculated as -SO_3_^-^) and proteins (calculated as N × 4.37); Table S2: ^13^C NMR assignments of bile salt, cholesterol, Trizma buffer, and TSP standard resonances; Figure S1: Viable cells (%) cultured for 6 h with polysaccharide-enriched fractions obtained from *S. latissima* (Sn_CaCl_2_, Ppt_CaCl_2_, F1, F2, and F3) at the concentrations of 25, 100, and 250 µg/mL, in the absence and presence of polymyxin B (PB). Culture medium alone (RPMI) was used as negative control. Lipopolysaccharide (LPS) and concanavalin A (ConA) were used as positive controls. Mean (±SD) values are represented. Different letters above the bars indicate statistically significant differences between compared groups (*p* < 0.05); Figure S2: Percentage of T cells activated by incubation with polysaccharide-enriched fractions obtained from *S. latissima* (Sn_CaCl_2_, Ppt_CaCl_2_, F1, F2, and F3) at the concentrations of 25, 100, and 250 µg/mL, in the absence and presence of polymyxin B (PB). Culture medium alone (RPMI) was used as negative control. Concanavalin A (ConA) was used as positive control. Mean (±SD) values are represented. Different letters above the bars indicate statistically significant differences between compared groups (*p* < 0.05); Figure S3: Representative ^13^C NMR spectrum of GDCA bile salt solution 50 mM (grey), GDCA bile salt solution 50 mM with labelled ^13^C_4_ Cholesterol 3.5 mM (black) in the presence of cationic resin colestipol 5 mg/mL (green) or polysaccharide fucoidan (F2) 5 mg/mL (blue); Figure S4: Gating strategy for flow cytometry analysis. Representative dot plots are presented showing the gating strategy for lymphocytes (FCS vs. SSC), non-aggregated cells (FCS-H vs. FCS-A), live cells (propidium iodide), B and T cells (CD3 vs. CD19), and activated cells (CD69 vs. CD3 and CD69 vs. CD19).
