# Supplementary material for: Water-Soluble Saccharina latissima Polysaccharides and Relation of Their Structural Characteristics with In Vitro Immunostimulatory and Hypocholesterolemic Activities
Source: Mar Drugs. 2023 Mar 16;21(3):183. doi: 10.3390/md21030183 (PMC10054259; doi:10.3390/md21030183)
Supplement: Supplementary file 1 [file marinedrugs-21-00183-s001.zip › marinedrugs-2224335-supplementary.pdf]

Supplementary material

Article

Water-soluble *Saccharina latissima* polysaccharides and relation of their structural characteristics with *in vitro* immunostimulatory and hypocholesterolemic activities

Ana S. P. Moreira [1], Diana Gaspar [1], Sónia S. Ferreira [1], Alexandra Correia [2,3], Manuel Vilanova [2,3], Marie-Mathilde Perrineau [4], Philip D. Kerrison [4,5], Claire M. M. Gachon [4,6], Maria Rosário Domingues [1,7], Manuel A. Coimbra [1], Filipe M. Coreta-Gomes [1,8] and Cláudia Nunes [9,*]

1 LAQV-REQUIMTE—Associated Laboratory for Green Chemistry of the Network of Chemistry and Technology, Department of Chemistry, University of Aveiro, Campus Universitário de Santiago, 3810-193 Aveiro, Portugal
2 i3S—Institute for Research and Innovation in Health and IBMC—Institute for Molecular and Cell Biology, University of Porto, 4200-135 Porto, Portugal
3 ICBAS — Instituto de Ciências Biomédicas de Abel Salazar, Universidade do Porto, 4050-313 Porto, Portugal
4 Scottish Association for Marine Sciences, Scottish Marine Institute, Oban PA37 1QA, UK
5 Hortimare BV, Altonstraat 25A, 1704 CC Heerhugowaard, The Netherlands
6 Unité Molécules de Communication et Adaptation des Micro-Organismes (UMR 7245), Muséum National d’Histoire Naturelle, Centre National de la Recherche Scientifique (CNRS), 75005 Paris, France
7 CESAM—Centre for Environmental and Marine Studies, Department of Chemistry, University of Aveiro, Campus Universitário de Santiago, 3810-193 Aveiro, Portugal
8 CQC-IMS — Coimbra Chemistry Centre, Institute of Molecular Sciences, University of Coimbra, 3004-535 Coimbra, Portugal
9 CICECO — Aveiro Institute of Materials, Department of Materials and Ceramic Engineering, University of Aveiro, Campus Universitário de Santiago, 3810-193 Aveiro, Portugal

* Correspondence: claudianunes@ua.pt

Supplementary Table S1. Content (%, w/w) of sulphur (S) and nitrogen (N) determined by elemental analysis, as well as sulphates (calculated as $-SO_3^-$) and proteins (calculated as N × 4.37).

**Sample**	**Elemental analysis**		**Sulphates (calculated as $-SO_3^-$)**	**Proteins (N × 4.37)**
	**S**	**N**		
Sn_$CaCl_2$	2.5 ± 0.03	2.6 ± 0.02	6.4 ± 0.1	11.2 ± 0.1
F1	-	0.6 ± 0.04	-	2.6 ± 0.2
F2	1.9 ± 0.3	4.7 ± 0.1	4.8 ± 0.4	20.7 ± 0.2
F3	5.7 ± 0.6	1.4 ± 0.01	14.3 ± 1.5	6.2 ± 0.02

Data are presented as mean ± standard deviation.

Supplementary Table S2. ^{13}C NMR assignments of bile salt, cholesterol, Trizma buffer, and TSP standard resonances.

ppm	Assignments	Molecular Structure
179.5	GDCA, C_{24} (quaternary carbon)	
179.4	GDCA, C_{26} (quaternary carbon)	
75.7	GDCA, C_{12} (tertiary carbon)	
74.1	GDCA, C_{3} (tertiary carbon)	
63.3	Trizma, C_{1}, C_{2}, C_{3} (methylene carbon)	
62.7	Trizma, C_{4} (tertiary carbon)	
50.6	GDCA, C_{14} (tertiary carbon)	
49.1	GDCA, C_{17} (tertiary carbon)	
49.0	GDCA, C_{13} (quaternary carbon)	
46.2	GDCA, C_{25} (methylene carbon)	
45.0	GDCA, C_{5} (tertiary carbon)	
44.3	Cholesterol, $^{13}C_{4}$ (tertiary carbon)	
38.9	GDCA, C_{8} (tertiary carbon)	
38.4	GDCA, C_{20} (tertiary carbon)	
38.2	GDCA, C_{1} (methylene carbon)	
38.1	GDCA, C_{4} (methylene carbon)	
36.8	GDCA, C_{10} (quaternary carbon)	
36.2	GDCA, C_{9} (tertiary carbon)	
35.3	GDCA, C_{23} (methylene carbon)	
34.3	GDCA, C_{22} (methylene carbon)	
32.1	GDCA, C_{2} (methylene carbon)	
31.2	GDCA, C_{11} (methylene carbon)	
30.4	GDCA, C_{16} (methylene carbon)	
30.0	GDCA, C_{6} (methylene carbon)	
29.0	GDCA, C_{7} (methylene carbon)	
26.6	GDCA, C_{15} (methylene carbon)	
25.8	GDCA, C_{19} (methyl carbon)	
19.5	GDCA, C_{21} (methyl carbon)	
15.4	GDCA, C_{18} (methyl carbon)	
0.0	TSP, C_{4}, C_{5}, C_{6} (methyl carbons)	

GDCA Bile Salt

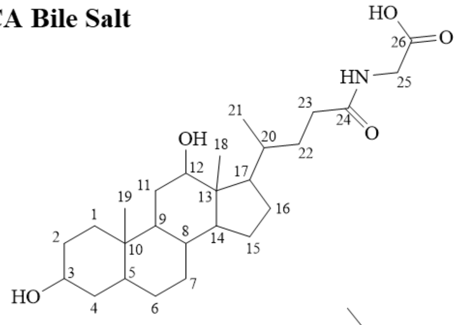

$^{13}C_4$-Cholesterol

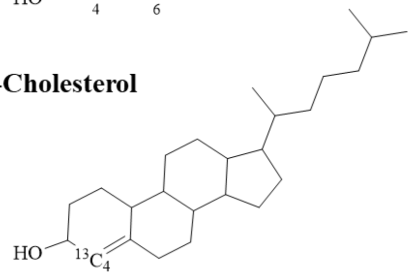

Trizma Buffer

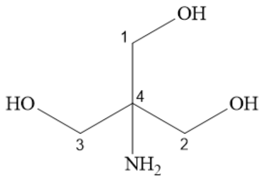

TSP standard

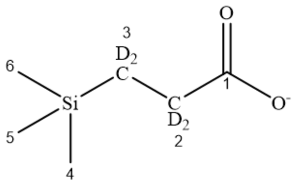

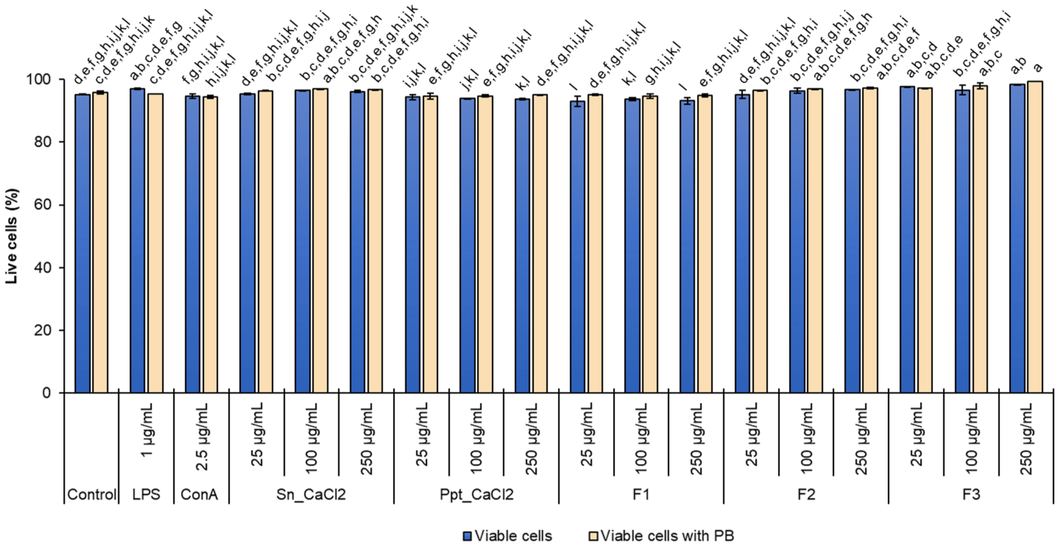

Supplementary Figure S1. Viable cells (%) cultured for 6 h with polysaccharide-enriched fractions obtained from *S. latissima* (Sn_$CaCl_2$, Ppt_$CaCl_2$, F1, F2, and F3) at the concentrations of 25, 100, and 250 µg/mL, in the absence and presence of polymyxin B (PB). Culture medium alone (RPMI) was used as negative control. Lipopolysaccharide (LPS) and concanavalin A (ConA) were used as positive controls. Mean (± SD) values are represented. Different letters above the bars indicate statistically significant differences between compared groups (p<0.05).

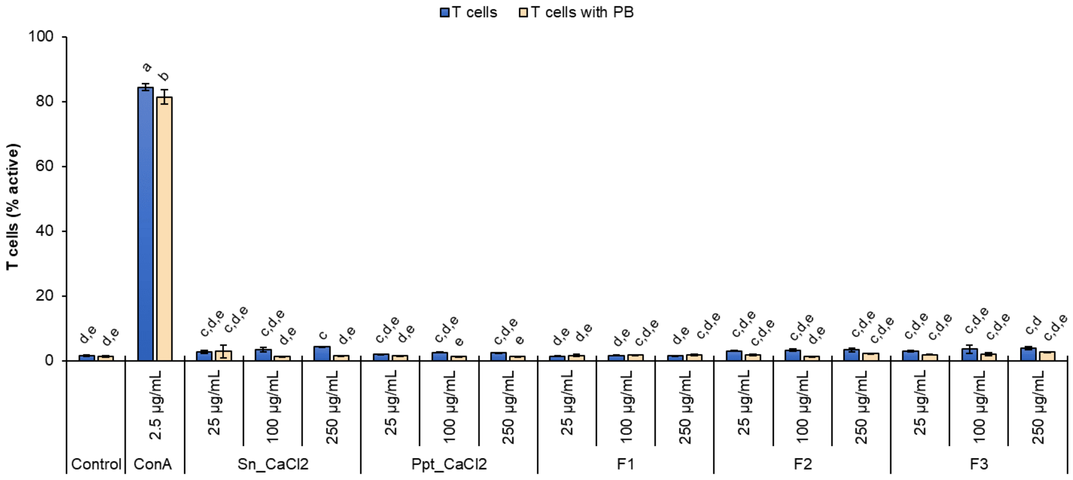

Supplementary Figure S2. Percentage of T cells activated by incubation with polysaccharide-enriched fractions obtained from *S. latissima* (Sn_$CaCl_2$, Ppt_$CaCl_2$, F1, F2, and F3) at the concentrations of 25, 100, and 250 µg/mL, in the absence and presence of polymyxin B (PB). Culture medium alone (RPMI) was used as negative control. Concanavalin A (ConA) was used as positive control. Mean (± SD) values are represented. Different letters above the bars indicate statistically significant differences between compared groups (p<0.05).

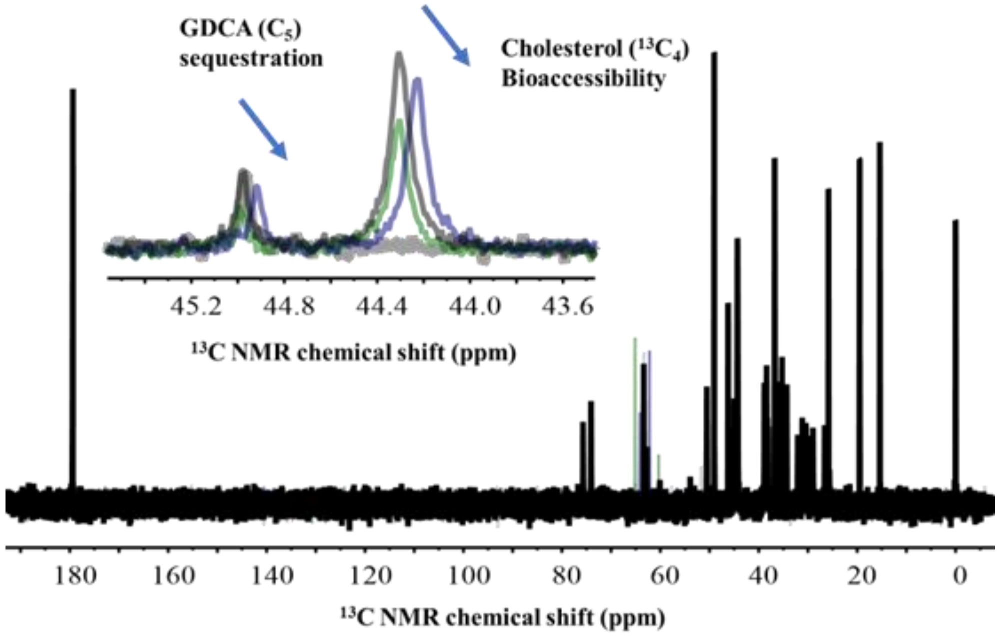

Supplementary Figure S3. Representative ^{13}C NMR spectrum of GDCA bile salt solution 50 mM (grey), GDCA bile salt solution 50 mM with labelled $^{13}C_4$ Cholesterol 3.5 mM (black) in the presence of cationic resin colestipol 5 mg/mL (green) or polysaccharide fucoidan (F2) 5 mg/mL (blue).

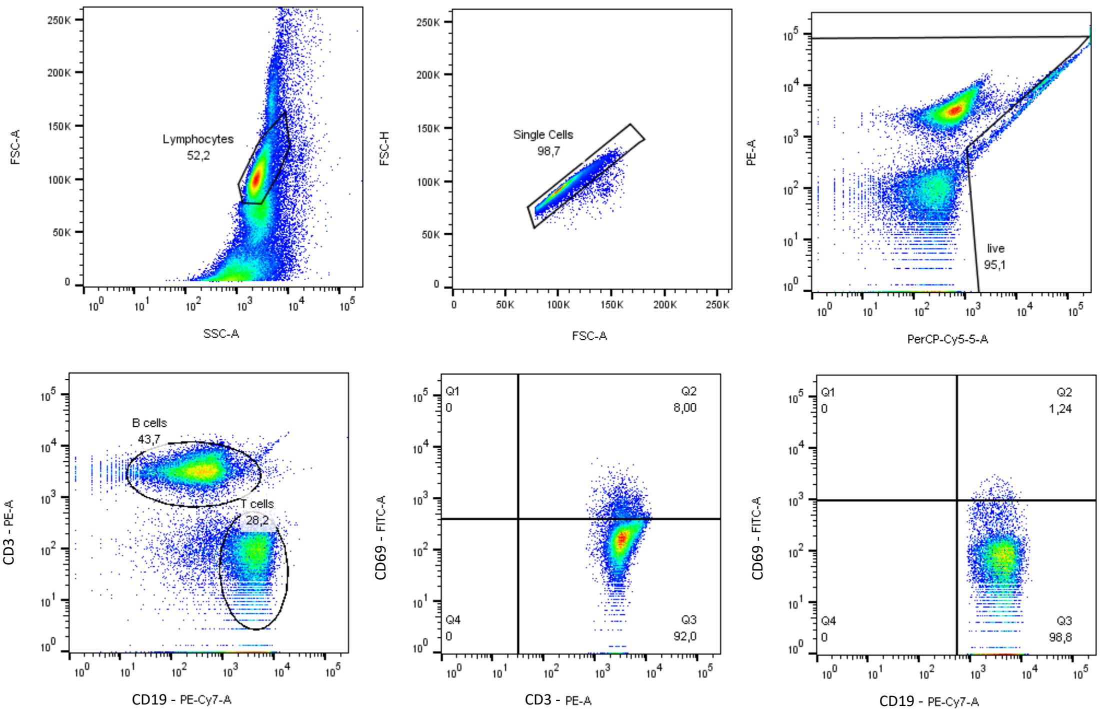

Supplementary Figure S4. Gating strategy for flow cytometry analysis. Representative dot plots are presented showing the gating strategy for lymphocytes (FCS vs SSC), non-aggregated cells (FCS-H vs FCS-A), live cells (propidium iodide), B and T cells (CD3 vs CD19), and activated cells (CD69 vs CD3 and CD69 vs CD19).
